# Registration Attendants Show Poor Readiness to Handle Advanced Care Planning Discussions

**DOI:** 10.1089/pmr.2021.0006

**Published:** 2021-11-22

**Authors:** Kevin D. Webster, Sabrina Webster, Suzanne Michelle Rhodes

**Affiliations:** ^1^ Diné College, School of Science, Technology, Engineering and Math, Tsaile, Arizona, USA.; ^2^ Planetary Science Institute, Tucson, Arizona, USA.; ^3^ Family Medicne Resident Physician, Aurora Health Care, Milwaukee, Wisconsin, USA; ^4^ Palliative and Emergency Medicine Physician, Flagstaff Medical Center, Flagstaff, Arizona, USA.

**Keywords:** advance directives, emergency department, palliative care

## Abstract

***Background*:** Emergency departments (ED) and other medical points of care are required to provide patients with advance directive (AD) information. Although many hospitals provide AD information in EDs, the comfort and preparation of the ED staff with this responsibility is unclear.

***Objective*:** To determine the attitudes, comfort levels, and prior training of ED staff with AD.

***Methods*:** The ED social workers, nurses, registration attendants, residents, and attending physicians at two academic hospitals completed a survey about their attitudes around, preparedness for, and experiences with advance care planning (ACP) discussions in the ED.

***Results*:** We received responses from 220 ED staff. Preparedness to discuss ACP with patients varied by profession. Eighty percent of social workers (*n* = 4/5) and 52% (*n* = 16/31) of attending physicians reported preparedness to handle ACP discussions. Registration attendants were the least prepared, and only 4% (*n* = 1/24) reported preparedness to discuss ACP. Attempts at ACP discussions with patients also differed by profession, with attending physicians being the most likely (77%, *n* = 24/31), whereas registration attendants were the least likely (8%, *n* = 2/24). Fifty-nine percent of surveyed staff (*n* = 130/220) believed that ACP was a component of emergency care, although only 13% (*n* = 29/220) had received training.

***Conclusion*:** The ED staff are in favor of ACP in the ED. Preparedness for, and attempts of ACP discussions with patients in the ED vary by profession. Attending physicians and social workers tend to be the most prepared, and they report the most frequent attempts at discussions with patients. Despite the fact that registration attendants are frequently tasked with asking about patient ADs, they show little confidence in asking about and discussing such matters. Our research indicates that registration attendants feel unprepared to guide discussions of ADs and should not do so without additional training.

## Introduction

Advance directives (AD) are documents that allow patients to direct their future care in the event that they do not have the capacity to do so. This is accomplished by selecting a decision maker (medical power of attorney) and/or directing the type of care they would want under specific circumstances via a living will. Many states also participate in the Physician Orders for Life-Sustaining Treatment (POLST) or the Medical Orders for Life-Sustaining Treatment (MOLST) programs that cover patient preferences at the end of life.^1^

The Patient Self-Determination Act (PSDA) of 1991 sought to improve rates of AD completion and availability,^2^ and many states have laws encouraging AD completion.^3^ Although the PSDA and other laws are not specific to Emergency Departments (EDs), many hospital systems stay in compliance by asking patients at the time of their registration in the ED whether they have an AD, would like a copy placed in the record, or would like information about ADs.^4^^,^^5^

It is important that the ED staff in charge of inquiring about a patient's AD status are prepared to address questions, assist with completion, and provide resources for patients who are interested. The ADs are recognized as an important component of patient care,^6^^,^^7^ and patient satisfaction with care can improve if treatment aligns with a patient's goals.^8^ Many patients experience their first encounter with ADs in the ED. As a result, ED patients tend to have low rates of AD completion (between 21% and 53%) and ADs are often not readily available (1–44%).^9^ These ED-particular circumstances enhance the need for effective training in AD among ED personnel.

Currently, ED staff at all levels may partake in advanced care planning (ACP) at different points in their care of the patient. Although well intentioned, it is not clear whether ED staff are comfortable or prepared for this role. For example, only 59% of EM residency programs include palliative care training.^10^ Past research has shown that ED doctors find it difficult to have ACP discussions in the ED, due to a lack of time, and the sometimes limited ability to access a patient's medical history.^11^^,^^12^

Physicians have also reported that communication with patients and patient complexity contributes to the challenges facing ACP discussions in the ED.^13^ Many institutions leave this task to ED registration clerks who are typically also responsible for obtaining demographic, insurance, and financial information as well as consent to treat. Comfort with this task and training across ED staff has not yet been explored extensively.

Efforts to improve access and availability should start with understanding the knowledge and attitudes of those commonly present when the task is completed. The attitudes and preparedness among ED staff may vary by position, and these factors are rarely accounted for in admissions practices. This study sought to identify the holes in the knowledge surrounding the current state and preparedness of ED staff in discussing ADs by developing and administering a survey to determine the attitudes, comfort, and prior training with ADs.

## Methods

### Setting

Surveys were distributed at two affiliated academic hospital EDs with two associated emergency medicine residencies at the University of Arizona in Tucson. The hospitals use a fee-for-service payment structure. The ED social workers, nurses, registration attendants, residents, and attending physicians were included. During the study period, both hospitals used Epic (Verona, WI) as the electronic medical record.

### Survey design

The survey was developed by an interdisciplinary team of emergency medicine, palliative medicine physicians, and geriatricians, an advanced practice RN, social workers, and a PhD in epidemiology. The survey was designed to assess the attitudes of all ED staff who may engage in discussions around ADs and ACP for ED patients.

Staff were asked about their knowledge of the PSDA, discussions of ACP with patients, and which staff members are best equipped to handle AD discussion. Before distribution, the survey was approved by the local institutional review board. The survey was piloted and reviewed for clarity, content, and wording. Although many states have POLST or MOLST programs, these were not studied specifically since Arizona was not participating at the time of the study.

The survey included yes–no responses, free response questions, and responses that captured patient attitudes on 5-point Likert scales. Free response answers were categorized as “yes” or “no” according the specific response. Examples of the survey coding used to do this are presented in Appendix Table A1.

### Distribution

The survey that was distributed via SurveyMonkey (www.surveymonkey.com) or a printed version was handed to participants. An introduction to the study, the review board approval, and consent were emailed to all subjects via SurveyMonkey. A reminder email was sent the next week. Subjects who had not responded were provided with a paper copy in the mailbox and a locked box to place completed surveys.

### Analysis

Survey results were analyzed by using STATA 13.0 and R.^14^ Response rates and descriptive statistics of demographic data were calculated through two primary methods. Survey uncertainty was calculated by using the standard error equation. We report uncertainty in the survey responses to 1 standard error. Chi-square (*χ*^2^) tests were used to analyze differences to survey responses by respondent class. *p*-Values <0.05 were considered significant.

## Results

Response rates varied by category, with registration attendants having the highest rates at 86% and nursing the lowest at 47%. Overall, 220 responses were analyzed, resulting in a 53% response rate (Table 1). The demographics of the survey respondents are recorded in Table 2. The majority of respondents are females in their 30s.

**Table 1.** Survey Response Rates by Job Title

Category	Sent	Response	%
Social work	7	5	71%
Resident	77	42	55%
Attending	55	31	56%
Nursing	246	118	48%
Registration	28	24	86%
Totals	413	220	53%

**Table 2.** Demographics

Position, *N* (%)	220 (100)
Attending	31 (14)
Nursing	118 (53)
Registration	24 (11)
Resident	42 (19)
Social Work	5 (2)
Age, median (IQR)	36 (30–43)
Attending	37 (35–46)
Nursing	38 (31–44)
Registration	39.5 (29–50)
Resident	29.5 (28–32)
Social Work	44 (36–50)
Sex, female, *N* (%)	147 (67)^a^
Attending	6 (19)
Nursing	96 (82)^a^
Registration	21 (88)
Resident	19 (45)
Social work	5 (100)

^a^ Numbers do not add up due to missing data.

IQR, interquartile range.

Only 21% (*n* = 45/220) of the staff surveyed were familiar with the PSDA. Once the act was explained, a majority (67%, *n* = 148/220) believed that this contributed to a better understanding of a patient's wishes at the end of life. Social workers and attending physicians had lower confidence that this practice improved patient care (attending physicians 48%, *n* = 15/31; social workers 40%, *n* = 2/5). The majority (72%, *n* = 158/220) of staff believed that the PSDA did improve documentation and availability of ADs. Among the ED staff, social workers (41%, *n* = 91/220) as well as doctors (attendings: 27%, *n* = 61; residents: 12%, *n* = 27, total: 40%) were viewed as the most appropriate specialties to discuss with AD with patients (Table 3).

**Table 3.** Responses to Select Survey Questions by Position in Emergency Department

	Affirmative responses, *n* (%)	Negative responses, *n* (%)	*p*
Do you know what the PSDA is?	45 (20)	173 (80)	0.01
Attending	7 (22)	24 (77)	
Nursing	28 (24)	89 (75)	
Registration	4 (17)	20 (83)	
Resident	3 (7)	39 (92)	
Social work	3 (60)	1 (20)	
Do you believe the PSDA contributes to a better understanding of a patient's preferences for end-of-life care?	148 (67)	68 (31)	0.08
Attending	15 (48)	15 (48)	
Nursing	81 (69)	36 (31)	
Registration	17 (71)	7 (29)	
Resident	33 (79)	8 (19)	
Social work	2 (40)	2 (40)	
Do you believe the PSDA contributes to better documentation and availability of ADs?	158 (72)	54 (25)	0.12
Attending	19 (61)	10 (32)	
Nursing	80 (68)	34 (29)	
Registration	19 (79)	4 (17)	
Resident	37 (88)	5 (12)	
Social work	3 (60)	1 (20)	
Have you ever attempted a discussion of ACP with a patient in the ED?	107 (49)	111 (50)	0.0001
Attending	24 (77)	7 (23)	
Nursing	46 (39)	71 (61)	
Registration	2 (8)	22 (92)	
Resident	32 (76)	10 (24)	
Social work	3 (60)	1 (20)	
Did you find the patient overall to be *(Main Exploration*)			
Interested and eager	9 (8)	NA	
Interested and willing	76 (71)	NA	
Uninterested and somewhat resistant	8 (8)	NA	
Openly resistant	1 (1)	NA	
Other	13 (12)	NA	
Do you see ACP as a component of emergency care?	130 (59)	19 (9)	
Attending	22 (71)	3 (9)	
Nursing	63 (53)	9 (8)	
Registration	10 (41)	4 (17)	
Resident	32 (76)	3 (7)	
Social work	3 (60)	0 (0)	
Have you received training in ACP?	29 (13)	126 (57)	0.005
Attending	7 (23)	18 (58)	
Nursing	9 (8)	68 (58)	
Registration	0 (0)	14 (58)	
Resident	11 (26)	25 (59)	
Social work	2 (40)	1 (20)	
Do you feel ED patients should be offered the opportunity to complete an AD or other components of ACP while in the ED?	183 (83)	30 (14)	0.05
Attending	26 (83)	5 (16)	
Nursing	99 (84)	16 (14)	
Registration	15 (62)	7 (29)	
Resident	39 (93)	2 (5)	
Social work	4 (80)	0 (0)	
Of all the ED staff, who do you think is the most appropriate to assist with AD completion and discussion (*main exploration*)?			
Attending	61 (27)	NA	
RN	8 (4)	NA	
Registration	5 (2)	NA	
Resident	27 (12)	NA	
Social work	91 (41)	NA	
Do you have an AD?	46 (21)	168 (76)	0.002
Attending	11 (35)	20 (65)	
Nursing	26 (22)	89 (75)	
Registration	1 (4)	21 (88)	
Resident	5 (12)	37 (88)	
Social work	3 (60)	1 (20)	

Percentages may not add to 100 due to nonresponse.

^*^ ACP, advance care planning; AD, advance directive; ED, emergency department; PSDA, Patient Self Determination Act.

**FIG. 1.**
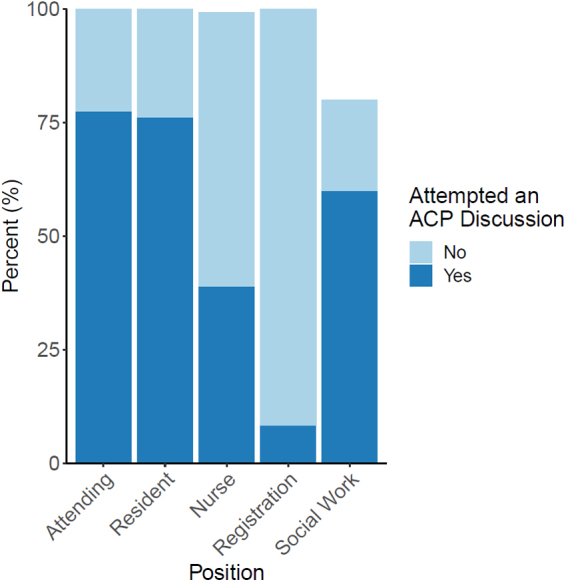
The distributions of whether health care professionals have attempted an ACP discussion broken down by their position. Note that bars may not be total 100% due to nonresponse. ACP, advance care planning.

Health care professionals differed in whether they had attempted an ACP discussion by their position (*χ*^2^ test, *χ*^2^ = 42.7, df = 3, *p* < 0.01) (Fig. 1). Attending physicians were the most likely to have discussed ACP (77% ± 8%, *n* = 24/31), followed by residents (76% ± 7%, *n* = 32/42); registration attendants were the least likely (8% ± 6%, *n* = 2/25). Men and women differed in whether they had attempted ACP discussions (men: 63% ± 6%, women: 41% ± 6%, *χ*^2^ test, *χ*^2^ = 8.7, df = 1, *p* < 0.01).

Nevertheless, 83% (183/220) surveyed believed that ACP was an important component of emergency care, although only 13% (29/220) had received training. Social workers were the most likely to report prior training, as opposed to registration attendants, who reported having no prior training. Most staff members believed in offering ED patients an opportunity to complete an AD (59%, *n* = 130/220).

**FIG. 2.**
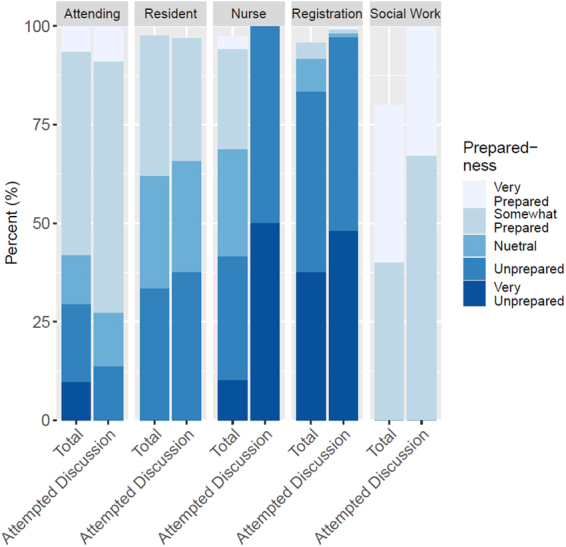
Providers' personal assessment of their preparedness to answer questions and assist with the completion of advance directives broken down by their position and whether they had attempted a discussion of the topic. Note that bars may not total 100% due to nonresponse.

Health care professionals differed in their preparedness to discuss ACP with their patients based on their profession (*χ*^2^ test, *χ*^2^ = 134.6, *df* = 12, *p* < 0.01) (Fig. 2). Social workers were the most prepared to handle ACP discussions (Prepared and somewhat prepared, 80% ± 15%, *n* = 4/5), but confidence in this response is limited by the low numbers of respondents (*n* = 5).

A majority of attending physicians (prepared and somewhat prepared, 58% ± 9%, *n* = 18/31), but a minority of residents (prepared and somewhat prepared, 36% ± 7%, *n* = 15/42) were equipped to handle ACP discussions with patients. In addition, registration attendants as a group were the least prepared (prepared and somewhat prepared, 4% ± 4%, *n* = 1/24).

The proportions of health care professionals differed in their preparedness to discuss ACP based on whether they had actually attempted discussions of ACP (Fig. 2). Attending physicians showed more preparedness if they had attempted ACP discussions with their patients (*χ*^2^ test, *χ*^2^ = 12.4, *df* = 4, *p* = 0.01). Conversely, registration attendants reported being less prepared to discuss ACP with patients if they had attempted a discussion (*χ*^2^ test, *χ*^2^ = 10.0, df = 4, *p* = 0.04) (Fig. 2).

**FIG. 3.**
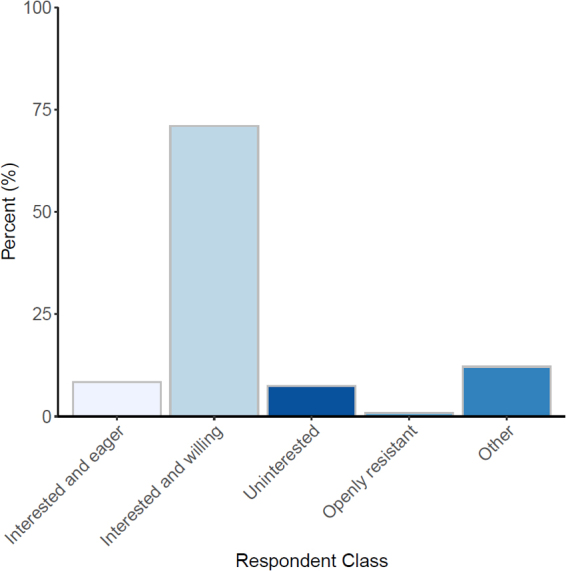
Patient attitudes toward discussing advance directives. Notice that most patients are “Interested and Willing.”

Men were more likely to report being more prepared than women to discuss ACP with their patients (*χ*^2^ test, *χ*^2^ = 12.2, df = 4, *p* = 0.02). Within a profession, however, men and women did not differ in the preparedness to discuss ACP with their patients (e.g., Attendings: *χ*^2^ test, *χ*^2^ = 3.2, df = 4, *p* = 0.52). The ED staff found that patients were generally interested and willing to discuss ACP (71% ± 4%, *n* = 76/107) compared with the rest of the attitudes (Fig. 3).

## Discussion

The ED staff are generally in favor of ACP in the ED. Attending physicians and social workers tend to see the highest value and report the most frequent attempts at discussions with patients. Our survey showed that 88% (*n* = 22/25) and 91% (*n* = 32/35) of participating attendings and residents thought ACP is important for patient care. In a previous survey, more than 90% of emergency physicians agreed that ACP is important for patient care,^6^ which is consistent with our results.

Only a few staff members had any training specific to discussing or completing ACP with patients. Further, with the exception of social workers, rates of AD administration by the medical staff were low overall (49%, *n* = 107/220), and low rates of AD administration have been correlated with AD completion among patients.^15^ Low responses are also not unique among surveys of ED staff and have been observed in prior studies.^16^

Many hospitals task registration attendants with inquiring as to whether patients have an AD or would like to place one on file. However, this group reports having no specific training at all (0%, *n* = 0/24) on any component of the discussion. Despite having to ask all patients about these documents or desire for more information, the survey results indicate that only 8% (*n* = 2/24) of registration attendants have had an AD discussion.

Although the PSDA was well intentioned, the PSDA has often resulted in staff who are the least familiar, and have the lowest levels of comfort and training being tasked with this important conversation. It is recognized that the intention is to gather documents and provide information rather than a full ACP discussion with registration; however, if such an important task is left to the person gathering address and insurance information in passing, this runs the risk of downplaying the importance of AD and is a missed opportunity to have an informed discussion. The survey results suggest that registration attendants need training in or should not interact with patients regarding this critical portion of their care.

The results of this survey suggest that social workers or attending physicians are the most appropriate professionals to perform ACP discussions in the ED. This is supported by the preparedness of people in these positions to handle AD and is reflected in the ED staff's views as to which position is the most appropriate to handle these discussions.

A plurality of the surveyed ED staff view social workers (41%, *n* = 91/220) as the profession the most appropriate to handle ACP discussions, a view that has been observed earlier.^17^ In our survey, social workers exhibited high preparedness (80% ± 15%) to discuss ACP with patients. This is in agreement with past research that shows that social workers are skilled at ACP discussions and spend more time discussing AD with patients than physicians or nurses.^18^

Overall, 52% (*n* = 16/31) of attending physicians felt prepared to handle AD discussions with patients. Previous studies have shown that patients want to speak with physicians in these settings.^19^ In some cases, attending physicians may not be able to have an ACP discussion with a patient, and a resident or social worker may be a good substitute. A minority of residents in this survey reported being prepared (36%, *n* = 15/42) to handle ACP discussions, and 76% (*n* = 32/42) had attempted a discussion.

Importantly, our results imply differing patterns about ACP preparedness among ED staff as a result of prior experience. Attending physicians and residents appear to gain preparedness in ACP discussions with their patients through practice rather than by formal means. We infer this from the fact that attending physicians reported a greater preparedness than residents. However, none of the attending physicians who felt very unprepared to discuss ACP with their patients actually attempted an ACP discussion. This suggests that upfront training in ACP discussion among physicians could result in more discussions by physicians.

In fact, resident physicians who have experienced direct training in ACP discussions felt more confident assisting patients in AD.^20^ In addition, after training, variations in resident physician confidence by training year were eliminated,^20^ again supporting our idea that improved training may help doctors gain confidence. In contrast, the lack of confidence among registration attendants supports the notion that they should limit their involvement in ACP discussions to ascertaining the presence or absence of ADs, unless they receive additional training in facilitating ACP. However, one potential repercussion of having registration attendants ask about ADs may cause patients to experience alarm fatigue.

Although most would agree that the ED is not the ideal setting for ACP, the reality for many patients leaves a few other points of contact with health care in which to have this discussion or comply with the PSDA. Many ED staff lack adequate training or comfort with AD discussions, and this has been noted in several studies.^21–23^ Another study found that only 4% of patients who self-reported having a health care proxy or living will have had this documented in the EMR.^24^

Despite the lack of training, our results indicate overall high acceptability of ACP in the ED among staff. However, this disagrees with a survey of Canadian ED physicians who preferred AD completion by the admitting services such as internal medicine.^16^ Previous studies have also cited a lack of time, quiet spaces, and a lack of familiarity as a barrier to having ACP discussions in the ED.^13^^,^^14^^,^^16^^,^^25^ Further research should address patient acceptability, feasibility, and potential training that may improve the effectiveness of commonly used models to address the PSDA.

The difference in whether men and women had attempted ACP discussions—and the result that men reported being more prepared than women to discuss ACP with their patients—appears to be a result of their chosen professions. Sixty-six percent of men in the survey were either attending physicians or residents, whereas only 17% of women in the survey were of the same professions, a discrepancy that has been observed earlier.^26^ Attending physicians and residents reported being more prepared than nurses and registration attendants. In contrast, within a chosen profession, men and women did not differ in the preparedness to discuss ACP with their patients.

Patients were perceived as generally interested and willing to discuss ACP with ED staff. The interestedness of patients seems to contradict with the reported overall low rates of AD completion by patients before presenting at the ED.^9^ The low AD completion rates suggest that patients should be approached about ACP by ED staff in pertinent situations. Further, social workers, attending physicians, and residents are generally thought to be the best equipped to handle ACP discussions.

This suggests that in cases that are likely to require ACP, time should be set aside for social workers, attending physicians, or residents to discuss these issues with patients depending on the suite of professionals handling the case. However, time has often been identified as a barrier to ACP discussions in the ED.^11^^,^^12^^,^^16^^,^^25^

This survey of two EDs within a single health system may have limited applicability to other EDs, however many of the trends in this study are present in other research. A larger survey with a greater response rate may be helpful in determining how applicable our results are across the field. Survey completion rates in this study were moderate for nurses, residents, and faculty and only a small number of social workers completed the survey despite their high completion rates.

More data, or field-wide meta-analyses, where appropriate, could elucidate the attitudes of ED staff. A complete qualitative analysis was beyond the scope of this study but may have allowed for a deeper understanding of attitudes. Lastly, it is not well described in the literature as to how most health systems accomplish the requirements of the PSDA. This survey is, therefore, most applicable to questioning about ACP in the ED.

## Conclusion

The ED staff are generally in favor of ACP in the ED. The different professions in the ED report different levels of preparedness to handle ED discussions. Social workers and attending physicians tend to see the highest value and report the most frequent attempts at discussions with patients. Despite the fact that registration attendants are frequently tasked with asking about a patient's AD, they show little confidence in asking about and discussing such matters.

As a result, registration attendants should not handle patient ACP discussions. Further, attending physicians and social workers appear the best equipped to handle ACP discussions with patients. Additional training in ACP discussions for attending physicians and residents may better prepare them to handle ACP discussions. Future research may address potential patient alarm fatigue, and feasibility training for ED staff that may improve the implementation of the PSDA.

## Abbreviations Used

ACP: advance care planning
AD: advance directive
ED: emergency departments
MOLST: Mechanical Orders for Life-Sustaining Treatment
POLST: Physicians Orders for Life-Sustaining Treatment
PSDA: Patient Self-Determination Act

## Appendix

**Appendix Table A1.** Do You Believe That the Patient Self-Determination Act Contributes to Better Documentation and Availability of Advance Directives? Why? Selected Quotes from 75 Responses

Yes	No
Attending: “Because it is a law it forces institutions to comply.” Nurse: “The patient receives information about advanced directives which can facilitate a family discussion about his/her wishes.” Resident: “It could just by simply having access to the materials. However, it could also be confusing and need more explanation—which is not always (although sometimes necessary) best done in the ED.” Registration: “It notify the care facility of pt wishes and who the pt chooses to decide of their healthcare choices.” Social Work: “Many patients want to complete these forms so if they are asked there is a greater likelihood that the forms will be completed.”	Attending: “It's just another check box that everyone ignores.” Nurse: “Registration ask if they have one but nobody follows up until we actually need it.” Registration: “Not all patients are able to understand the information given to them or are explained to them.” Resident: “This is probably something that should be done sooner rather than when a patient comes in acutely ill.” Social Work: “Because I think staff do not have a good understanding of explaining advanced directives, so they just parrot back the language on the forms they need the patient to sign.”
	Do you see advance care planning as a component of emergency care? Why or why not? Selected quotes from 105 responses	

Yes	No
Attending: “Necessary discussion since some of our patients are at the end of their lives.” Resident: “People usually do not think about those issues until they are sick and in the ED is a good place to start the conversation before things get worse.” Nurse: “When a patient is critically ill, it is important to understand what they want and to provide patient centered care. ACP is another way to provide patient centered care.” Registration: “Without certain documents on file all care will continue, even if it is against patients' wishes. These documents being completed here would benefit the patient's families and only make the process that much smoother.” Social Work: “For people experiencing a significant health crisis, a visit to the ED tends to bring forth thoughts of one's own mortality. Sometimes it is a visiting family member that brings it up. Regardless, it's an excellent opportunity to have what is ordinarily a difficult discussion to initiate.”	Attending: “Ideally I would like for it to be discussed before ED presentation with ample time to discuss.” Resident: “Though I feel with patient facility better equipped and with more time to discuss.” Nurse: “We are here to save lives and send upstairs. They can do that upstairs.” Registration “Because who ever plans to go to the ED.”
	Do you feel Emergency Department patients should be offered the opportunity to complete an advance directive or complete other elements of advance care planning while in the ED? Why or why not? Select quotes from 139 responses	

Yes	No
Attending: “I do not think this should be required of the ED physician, but would be appropriate as part of their overall visit.” Resident: “because its the only place some of our patients interact with the healthcare system, and they can say no if they don't want to discuss.” Nurse: “But who is going to facilitate this? Nursing won't be able to fit in in. SW is already stretched.” Registration “Yes, but with a social or case worker.” Social Work: “Because it is an opportunity to discuss patients wishes before admission, and could prevent an unwanted medical intervention and could prevent future suffering for patients and families.”	Attending: “limited ED staff, resources.” Resident: “Not advanced directives. They are too long and complicated and require too much consideration. The POLST form maybe, but not even sure that's appropriate to fill out in the ED.” Nurse: “This ideally should be done in the outpatient primary care setting or inpatient; the demands on ED staff are already exceeding reasonable capacity.” Registration: “Patients coming into the are no feeling well and are not really interested or paying attention to the information given.”

ACP, advance care planning; ED, emergency department; POLST, Physicians Orders for Life-Sustaining Treatment.
